# Three years into the pandemic: results of the longitudinal German COPSY study on youth mental health and health-related quality of life

**DOI:** 10.3389/fpubh.2023.1129073

**Published:** 2023-06-15

**Authors:** Ulrike Ravens-Sieberer, Janine Devine, Ann-Kathrin Napp, Anne Kaman, Lynn Saftig, Martha Gilbert, Franziska Reiß, Constanze Löffler, Anja Miriam Simon, Klaus Hurrelmann, Sabine Walper, Robert Schlack, Heike Hölling, Lothar Heinz Wieler, Michael Erhart

**Affiliations:** ^1^Department of Child and Adolescent Psychiatry, Psychotherapy, and Psychosomatics, University Medical Center Hamburg-Eppendorf, Hamburg, Germany; ^2^Infratest dimap, Berlin, Germany; ^3^Hertie School, Berlin, Germany; ^4^German Youth Institute, Munich, Germany; ^5^Department for Epidemiology and Health Monitoring, Robert Koch Institute, Berlin, Germany; ^6^Research Cluster Digital Global Public Health, Hasso-Plattner-Institute, University of Potsdam, Potsdam, Germany; ^7^Department of Public Health, Alice Salomon University of Applied Sciences, Berlin, Germany; ^8^Department of Psychology, Apollon University of Applied Sciences, Bremen, Germany

**Keywords:** mental health, children and adolescents, longitudinal, COVID-19, pandemic

## Abstract

**Purpose:**

For the past three years, the German longitudinal COPSY (***CO**VID-19 and **PSY**chological Health*) study has monitored changes in health-related quality of life (HRQoL) and the mental health of children and adolescents during the COVID-19 pandemic.

**Methods:**

A nationwide, population-based survey was conducted in May–June 2020 (W1), December 2020–January 2021 (W2), September–October 2021 (W3), February 2022 (W4), and September–October 2022 (W5). In total, *n* = 2,471 children and adolescents aged 7–17 years (*n* = 1,673 aged 11–17 years with self-reports) were assessed using internationally established and validated measures of HRQoL (KIDSCREEN-10), mental health problems (SDQ), anxiety (SCARED), depressive symptoms (CES-DC, PHQ-2), psychosomatic complaints (HBSC-SCL), and fear about the future (DFS-K). Findings were compared to prepandemic population-based data.

**Results:**

While the prevalence of low HRQoL increased from 15% prepandemic to 48% at W2, it improved to 27% at W5. Similarly, overall mental health problems rose from 18% prepandemic to W1 through W2 (30–31%), and since then slowly declined (W3: 27%, W4: 29%, W5: 23%). Anxiety doubled from 15% prepandemic to 30% in W2 and declined to 25% (W5) since then. Depressive symptoms increased from 15%/10% (CES-DC/PHQ-2) prepandemic to 24%/15% in W2, and slowly decreased to 14%/9% in W5. Psychosomatic complaints are across all waves still on the rise. 32–44% of the youth expressed fears related to other current crises.

**Conclusion:**

Mental health of the youth improved in year 3 of the pandemic, but is still lower than before the pandemic.

## 1. Introduction

Three years into the COVID-19 pandemic, empirical evidence has accumulated that the pandemic has posed a substantial mental health burden on children and adolescents in Germany and worldwide. Systematic reviews summarizing studies from 2020 until spring 2021 conclude that mental health has deteriorated, particularly at the start of the pandemic (1–7). However, one recent meta-analysis showed heterogenous results (8). The nationwide, longitudinal COPSY (***CO**VID-19 and **PSY**chological Health*) study (9) has collected data since the very start of the pandemic, i.e., for 3 years, including the latest data collections in 2022. It found that the prevalence of low Health-Related Quality of Life (HRQoL), mental health problems, and anxiety has been elevated over the course of the first three survey waves in 2020 and 2021 compared to prepandemic data (10, 11). In the first wave of the COPSY study, 40% of children and adolescents reported a reduced HRQoL. The second survey wave showed peak levels of reduced HRQoL and of depression and anxiety symptoms, affecting roughly 30% of the participants. Findings of the third wave indicated slight improvements in mental health and HRQoL.

Several studies showed that the prevalence of low HRQoL significantly increased in 2020 (7, 12–15), continued to be low in winter 2020 (9) and spring 2021 (16) and only improved in autumn 2021 (13). Despite this development, some children benefited from more free time and increased time with family during the lockdown in April 2020 (12).

Several longitudinal studies (17, 18) found that mental health problems increased during 2020. Some studies showed that mental health problems peaked in times of lockdowns and school closures [e.g., (19–21)]. An Australian study showed that mental health worsened with the length of lockdowns (22), while a study from Japan reported that the length of school closings was not predictive of mental health (23). Some studies even found an improvement in the well-being of some children during the early lockdown (24, 25). Further, mental health problems slightly improved in autumn 2021 (26).

Two reviews from 2020 described an average doubling of symptoms of anxiety (21%, 26%) and depression (25%, 29%) during the pandemic compared to the prepandemic period (7, 14). Anxiety peaks differ across countries between March to May 2020 (27) (New York, NY) (20) and summer 2021 (United Kingdom, UK) (28). Interestingly, studies from the UK (29) and China (30, 31) report less anxiety during home confinement (29, 31) and a decrease in anxiety from May 2020 to May 2021 (30). Such a recovery effect was also shown in the United States (US) (20) and in a Southern European study (27), but it remained unclear whether the recovery could fully or in part be explained by missing prepandemic data.

Similarly, most studies described an initial increase in depressive symptoms across countries including the Netherlands (17), the US (32), and Australia (21, 33–35). Depressive symptoms either continued to increase through May 2021 (17) or remained stable at a slightly higher level than before the pandemic (26). Only one study from New York reported a recovery effect for depression (20), but again whether full or partial remained unclear.

Another important finding that has been reported by several studies is that specific subgroups of youth (e.g., with previously existing mental health problems, a lower educational level or low socioeconomic status of their parents, with a migration background, limited living space) were particularly vulnerable with respect to their mental health during the pandemic (13, 19, 22, 35–37). On the other hand, children and adolescents with resources like family and social support and an optimistic attitude seem to cope better with the pandemic, i.e., tend to be healthier mentally (9), which has been shown prepandemically as well (38).

From the vastly growing body of research, we learn about mental health trajectories of children and adolescents during the pandemic, although most studies only cover the early years of the pandemic, from 2020 to summer 2021, and study results are mixed. In Germany, there was no lockdown in 2022, the third year of the pandemic, restrictions were loosened further and the COVID-19 infection spread through the country. Most of the 12- to 17-year-olds were either recovered or vaccinated (74.4%) as of November 2022. For 5- to 11-year-olds, the vaccination rate was 22.3% (39, 40). Children’s COVID-19 symptoms were generally mild and the physical health of children was on average not impacted severely. However, some studies [e.g., (18)] reported an increase in overall somatic health complaints in a three-wave representative Norwegian longitudinal study of adolescents. In the third year of the pandemic families also faced new crises such as the war in Ukraine, inflation, climate change, and in Germany worries about an energy crisis.

The aims of this study are as follows:

(1) To investigate how childrens’ and adolescents’ HRQoL and mental health in Germany changed during the past three pandemic years compared to prepandemic data. We hypothesize that the mental burden of the youth declined in the third year of the pandemic due to loosened restrictions and an ongoing adaptation process.
(2) To examine which children are still at risk or more resourceful than others. We expect to replicate our previous results (9) that socially disadvantaged children are still particularly vulnerable and that resourceful children are still mentally healthier in year three.
(3) To explore whether the pandemic still negatively impacts the youth in autumn 2022 or whether new crises (e.g., the climate crisis or the Ukraine war etc.) pose a greater burden. We hypothesize that children and adolescents worry more about new crises than the pandemic now.

## 2. Materials and methods

### 2.1. Study design and sample

The German COPSY study is one of the first population-based longitudinal studies to monitor HRQoL and mental health in children and adolescents during the COVID-19 pandemic in Germany and was conducted in five survey waves. Wave 1 (W1, May–June 2020) took place when Germany was under a partial lockdown, Wave 2 (W2, December 2020–January 2021) was conducted during a nationwide lockdown, Wave 3 (W3, September–October 2021) was undertaken after a summer with low infection rates and loosened restrictions, Wave 4 (W4) was at the end of winter (February 2022), when there were still regulations for private gatherings, and Wave 5 (W5) in autumn 2022 (September–October 2022) with only minimal restrictions in place.

For the COPSY study, families were invited via email to participate in a nationwide online survey which used quota sampling. This method helped to ensure that the sample reflected the sociodemographic characteristics of the German population. The initial participation rate at W1 was 45.8%. The sample sizes of W1-W3, as well as the design and findings of the first three waves, have been described elsewhere (26). Families who had previously participated in the COPSY study were re-invited to each follow-up. To compensate for drop-outs due to aging additional families were recruited, so that sociodemographic representativeness and comparability across all five waves was ensured [please see references (13, 26) for exemplary flowcharts of the sampling of Waves 1 to 3]. Parents provided information on their children and adolescents aged 7 to 17 years, and children and adolescents aged 11 to 17 years were asked themselves. In total, *n* = 2,471 families participated in at least one wave of the COPSY study. Samples sizes across all waves ranged between n = 1,586 to n = 1,618 parent-reports and n = 1,040 to n = 1,139 self-reports. Sample sizes and sociodemographic data of Waves 1 to 3 have been described elsewhere (26). Samples of Wave 4 and 5, which are the focus of this manuscript, consisted of n = 1,116 self-reports and n = 1,668 parent-reports (Wave 4) and n = 1,085 self-reports and n = 1,701 parent-reports (Wave 5). The range of response rates across all waves was 80.3 to 86.1% with a response rate for participation in all waves (W1-W5) of 86.1%.

**Figure 1:**
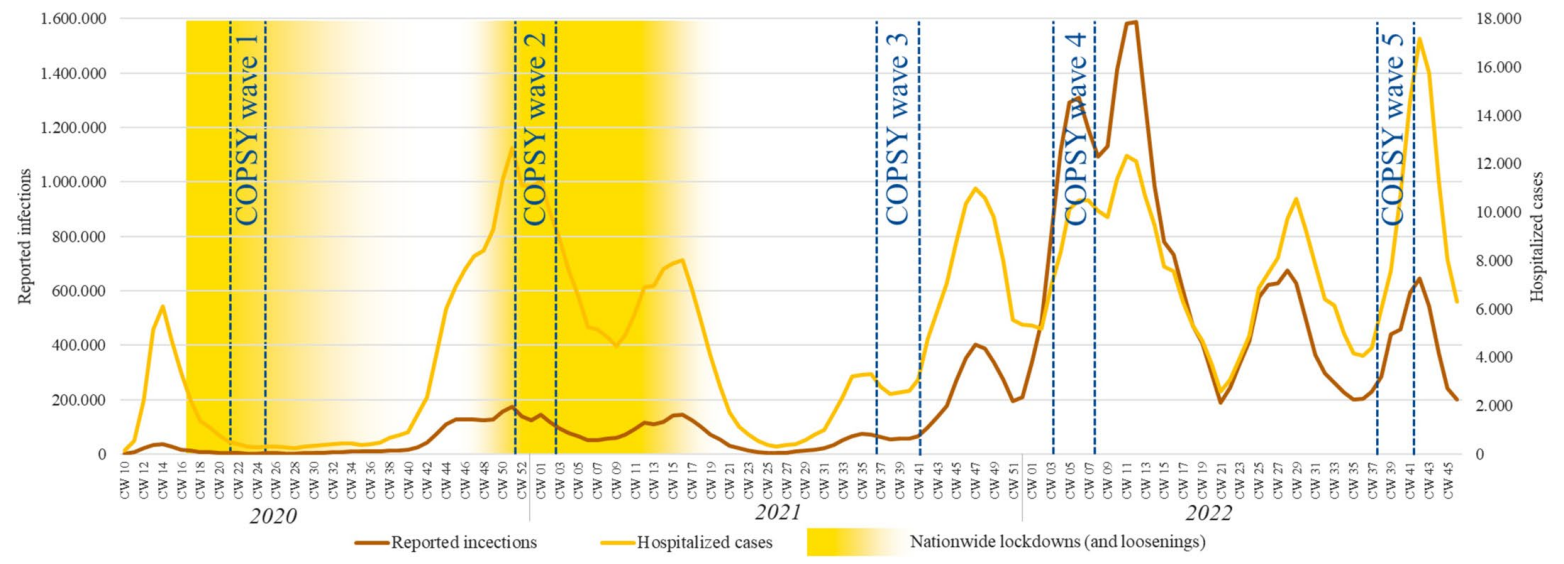
Timeline of the survey periods for COPSY W1 to W5 in relation to the infection and hospitalization rates in Germany.

See Figure 1 for the timeline of the COPSY waves in relation to the infection and hospitalization rates in Germany. It should be noted that the survey waves of the COPSY study should not be confused with COVID-19 infection waves, as epidemiologists have recently defined nine pandemic phases based on infection waves (40).

**Figure 2:**
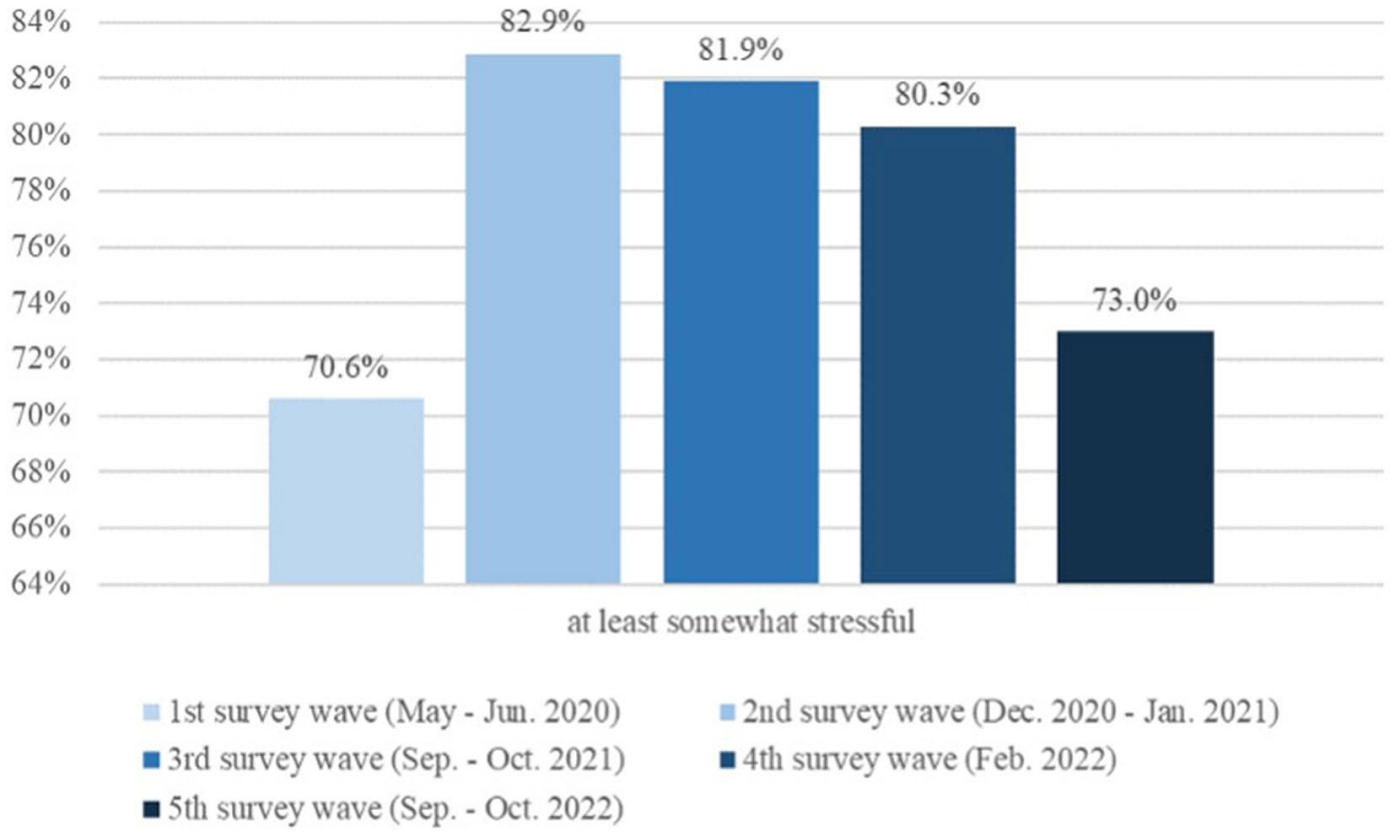
Perceived burden of the pandemic among children and adolescents 11–17 years old.

The data of each of the five waves was weighted in order to adjust the samples of the single survey waves to the sociodemographic characteristics of the German population (according to the 2018 Microcensus). Written informed consent to participate in this study was provided by the participants’ legal guardian/next kin. The COPSY study was approved by the Local Psychological Ethics Committee (LPEK-0151) and the Commissioner for Data Protection of the University of Hamburg.

For comparison with the HRQoL and mental health of children and adolescents prior to the pandemic, data of *n* = 1,020 participants (aged 11 to 17 years) from the nationally representative longitudinal BELLA study (*Behaviour and Well-being of Children and Adolescents in Germany;* (10), conducted between 2014 and 2017 was used. Prepandemic data on psychosomatic complaints of *n* = 1,073 children and adolescents, aged 11, 13, and 15 years, was taken from the German HBSC study (*Health Behaviour in School-aged Children;* (41)), which took place between 2017 and 2018.

### 2.2. Measures

#### 2.2.1. Sociodemographics

The survey covered questions on age, gender, education, living space, single-parenting and migration background.

#### 2.2.2. Health-related quality of life and the burden of COVID-19

For measuring health-related quality of life (HRQoL), the internationally established and widely used self-reported KIDSCREEN-10 Index (42) was used to classify children and adolescents as low, normal or high with respect to their HRQoL, compared to reference data from the national BELLA study. This instrument has proven to be valid and reliable in numerous studies (43–45). Normal HRQoL was defined as M_BELLA_+/-1SD_BELLA_.

The survey also included questions about an COVID-19 infection, child and family vaccination status, and whether a family member had died from COVID-19. The perceived burden of the pandemic was surveyed using two questions (13).

#### 2.2.3. Mental health in children and adolescents

Mental health problems were assessed with the internationally well-established Strengths and Difficulties Questionnaire [SDQ; (46)], including the problem scales: emotional problems, conduct problems, hyperactivity, and peer problems with five items each. By summing all 20 items of those subscales a *total difficulties* score can be generated. Higher scores indicate more severe problems. Using established cut-offs (47), participants were divided into two groups: those *with* and those *without* mental health problems (i.e., *abnormal/borderline* vs. *normal*).

Anxiety was assessed using the general anxiety symptom subscale of the Screen for Child Anxiety Related Disorders [SCARED; (48)]. A sum score of the nine items was calculated, with higher scores indicating more severe anxiety. Groups of participants *with* versus *without* anxiety were created using the established cut-off (48).

Depression was measured using the seven symptom items of the German version of the Center for Epidemiological Studies Depression Scale (CES-DC), which have been previously used in the BELLA study (49, 50). In addition, the Patient Health Questionnaire [PHQ-2; (51, 52)] was used. While the CES-DC measures impairment due to depressive symptoms within the past week, the two items of the PHQ-2 ask about core symptoms of major depressive disorder. A higher sum score indicates more severe depressive symptoms according to PHQ-2 and higher impairment by depressive symptoms according to CES-DC. Validated cut-offs were applied to categorize participants *with* and those *without* noticeable depressive symptoms (51, 53).

Further, the self-reported HBSC symptom checklist [HBSC-SCL; (54)], which is a longstanding validated instrument in international studies, was administered to measure the frequencies of psychosomatic complaints. Participants were divided into groups of subjects who experienced each psychosomatic symptom *at least once per week* vs. those who experienced it *less frequently*.

Additional questions were used to assess young people’s concerns about other current crises, such as the war in Ukraine, climate change, and the energy crisis. Those were rated on a 5-Point-Likert scale from *not at all* to *very* worried. In addition, a short version of the Dark Future Scale for Children [DFS-K; (55, 56)], measuring the tendency to think about the future with anxiety and uncertainty was administered to assess future anxiety and future worries in the context of different current crises. Regarding to Zaleski (56), future anxiety is defined as a “fear of future events and a feeling that dangerous or adverse changes may occur in the future.” A pandemic related version of the DFS-K was first administered in a sample of school students, where acceptable Cronbach’s Alpha (0.76), a Retest-correlation of 0.34 and content as well as construct validity are reported (55). All HRQoL and mental health measures (KIDSCREEN-10 Index, SDQ, SCARED, CES-DC, HBSC-SCL PHQ-2) are internationally validated and have good psychometric properties, which have been described previously (26).

#### 2.2.4. Personal, family, and social resources

The Personal Resources Scale [PRS; (57)] was administered to assess personal resources, such as problem-solving skills and optimism of children and adolescents. Perceived social support was measured with four items of the Social Support Scale [SSS; (58, 59)]. Four items from the Cohesion subscale of the Family Climate Scale (FCS) were used to assess family climate (60). Sum scores were calculated for all three scales (PRS, SSS, FCS) with higher values indicating more pronounced resources (57, 58, 60). Respondents with scores below the 20^th^ percentile in the prepandemic BELLA study were considered as having deficits in that particular resource.

### 2.3. Data analysis – change in HRQoL and mental health

First, the reported burden of the pandemic on children and adolescents was compared across the five COPSY waves. Second, the proportions of youth displaying impaired HRQoL, mental health problems, anxiety, depressive symptoms, and psychosomatic complaints were compared across all measurement points. Age- and gender-adjusted proportions were calculated using logistic regression models for each outcome. Prepandemic population-based data available from the BELLA study (10) and the HBSC study (41) were used to compare HRQoL and mental health outcomes. Chi-square tests and effect sizes (Phi coefficient ϕ, and Cramer’s V, resp. with 0.10 indicating a small, 0.30 a medium, and 0.50 a strong effect) were calculated for comparisons across the waves and for group comparisons. Gender differences were expressed as risk ratios (RR) for girls. The chi-square tests as well as the ϕ and V statistics for comparisons across the COPSY waves do not take into account that the majority of measurements represent repeated measures of the same respondents. This led to a lower statistical power in order to detect differences across the COPSY waves. The results of the Dark Future Scale and young people’s concerns about other recent crises were reported as descriptive statistics.

Additional logistic regression models were conducted to examine the association between risk group and resource cluster and the main COPSY outcomes (low HRQoL, mental health problems, anxiety, and depressive symptoms). All analyses were controlled for age and gender. Last logistic regression analysis were run to test the association between single-parenting and the above mentioned outcomes.

Alpha-error level was not adjusted because the outcomes were correlated across and within waves, the analyses thus cannot be considered as independent testing of the same hypothesis.

Prior to analyzing the data, a power analysis was conducted using G-Power (Version 3.1). The minimum sample size based on a statistical significance of *p*(*α*) < 0.05 and a power of *p* (1−*ß*) = 0.8 for a medium effect (*w* = 0.3) between waves according to age groups (7–10 years, 11–13 years, 14–17 years) and female vs. male was *n* = 88.

## 3. Results

### 3.1. Sociodemographics

In total, across all five survey waves of the COPSY study, *n* = 2,471 families with children and adolescents aged 7–17 years (*M* = 13.09, *SD* = 3.83, 50.1% female) completed parent reports. Self-reports were completed by *n* = 1,673 children and adolescents from age 11–17 (*M* = 15.23, *SD* = 2.56, 52.1% female). Sociodemographic characteristics are reported in Table 1 and Figure 2.

**Table 1:** Sociodemographic characteristics of the COPSY sample.

	Children and adolescents aged 7–17 years (parent reports) (*n* = 2,471)		Children and adolescents aged 11–17 years (self-reports) (*n* = 1,673)	
	*n* (%)	*M (SD)*	*n* (%)	*M (SD)*
Age^a^		13.09 (3.83)		15.23 (2.56)
7–10 years	775 (31.4)		–	
11–13 years	512 (20.7)		494 (29.5)	
14–17 years^b^	1,184 (47.9)		1,179 (70.5)	
Gender				
Male	1,221 (49.4)		792 (47.3)	
Female	1,239 (50.1)		872 (52.1)	
Other	10 (0.4)		9 (0.5)	
Age of the parent^a^		44.28 (7.57)		46.34 (7.22)
Gender of the parent				
Male	1,076 (43.5)		741 (44.3)	
Female	1,392 (56.3)		930 (55.6)	
Other	3 (0.1)		2 (0.1)	
Migration background				
No	2,033 (82.9)		1,376 (82.9)	
Yes	419 (17.1)		283 (17.1)	
Parental education				
Low	355 (14.4)		272 (16.3)	
Medium	1,397 (56.5)		945 (56.5)	
High	668 (27.0)		417 (24.9)	
No information	51 (2.1)		39 (2.3)	
Single parent				
No	2,018 (81.7)		1,338 (80.0)	
Yes	453 (18.3)		335 (20.0)	
Occupational status				
Full-time employed	1,281 (51.8)		892 (53.3)	
Part-time employed	754 (30.5)		486 (29.0)	
Self-employed	101 (4.1)		71 (4.2)	
Other employment	40 (1.6)		29 (1.7)	
Stay-at-home parent	147 (5.9)		96 (5.7)	
Retiree/pensioner	59 (2.4)		47 (2.8)	
On parental leave	33 (1.3)		13 (0.8)	
Unemployed	56 (2.3)		39 (2.3)	
COVID-19 infection^c^				
A family member has been infected	1,458 (85.7)		946 (84.0)	
The child has been infected	1,056 (62.1)		678 (60.2)	
A relative has died of COVID-19	156 (9.2)		109 (9.7)	

Unweighted data. M = mean; SD = standard deviation. ^a^Age at the latest time of participation. ^b^Some adolescents had already turned 18 when they participated in the survey but were included in the age group of 14- to 17-year-olds at W5. ^c^Any previous confirmed infection with the coronavirus according to parental report at W5.

Parents who participated in all five surveys were less likely to have a migration background (14.0% vs. 18.4%; *V* = 0.06), were more likely to have a lower level of education (18.7% vs. 12.9%; *V* = 0.09) and were on average 3.3 years older (*d* = 0.44) than those who only responded to 1–4 Waves. No other significant differences in sociodemographic or mental health-related variables were found.

### 3.2. Changes in HRQoL during the pandemic

After the initial increase in perceived burden of the pandemic (from W1: 70.6% to W2: 82.9%) in children and adolescents aged 11 to 17, the burden decreased between W3 and W4 and W5, i.e., changing from 81.9% in fall 2021 to 80.3% in February 2022 and most recently falling to 73.0% in fall 2022 (*p* < 0.001, ϕ = 0.08) (see Figure 2).

**Figure 3:**
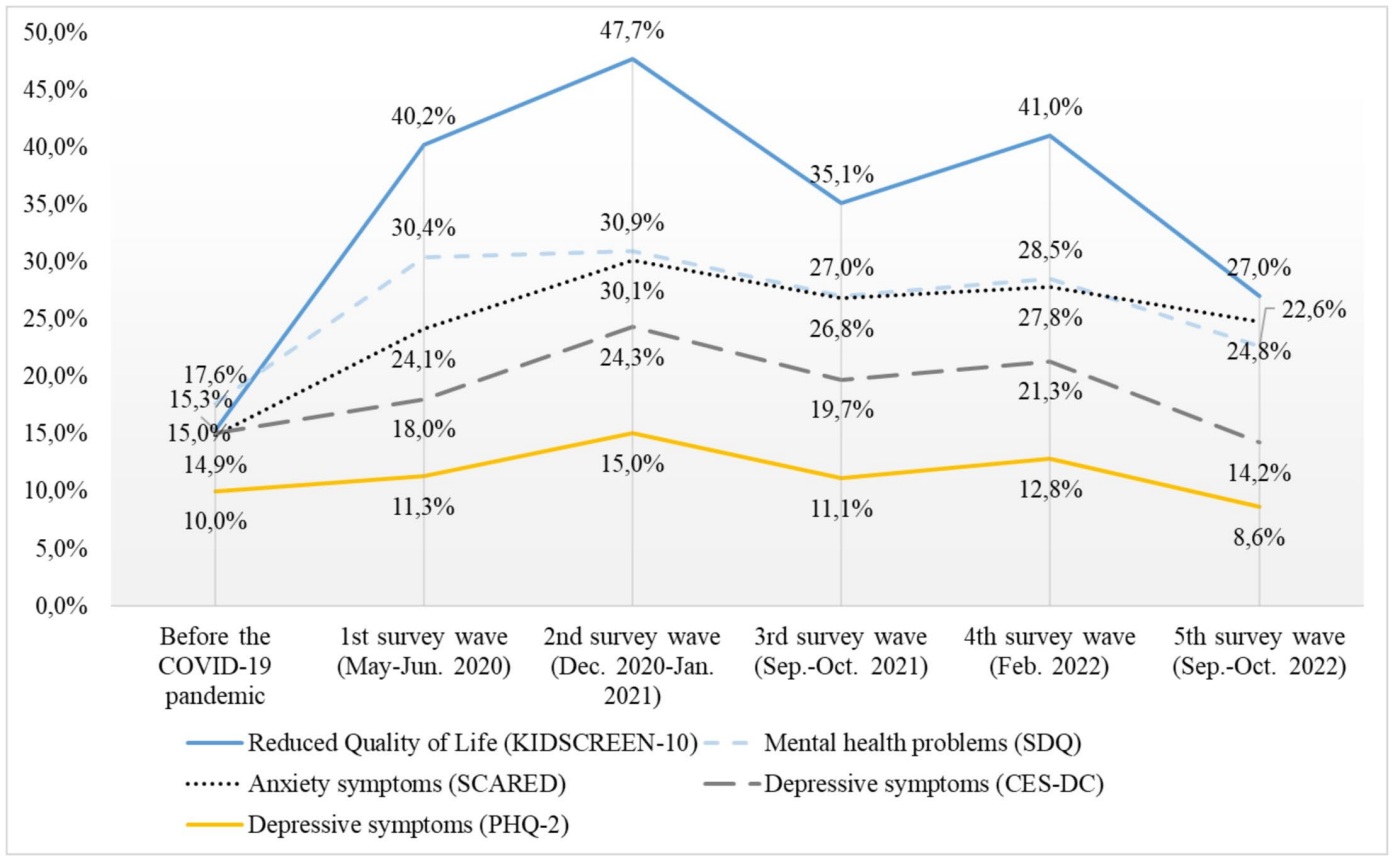
Mental health of children and adolescents aged 7–17 years from 2020 to 2022.

After the initial significant increases of the prevalence of low HRQoL from prepandemic 15.3% to 40.2% (W1) and 47.7% (W2) in 2020, it significantly dropped in spring 2021 to 35.1% (W3), increased in autumn of that year back up to 41.0% (W4), and most recently decreased to 27.0% (*p* < 0.001, ϕ = 0.14). This latest figure is still higher than prepandemic rates, but lower than at the start of 2020. The prevalence of low HRQoL at W5 is now less than twice as high as the prepandemic rate (see Figure 3). An analysis stratified by gender revealed that girls had a two-fold higher risk (Risk Ratio, RR) of low HRQoL compared to boys in prepandemic times, the RR decreased to 1.2 and 0.9 during the pandemic.

### 3.3. Changes in mental health during the pandemic

After the initial worsening of overall mental health problems (SDQ) in 2020 (from prepandemic: 17.6% to W1: 30.4%, W2: 30.9%), and a slight but non-significant decrease in mental health problems in Wave 3 (27.0% winter 2020), *via* 28.5% (W4) the downwards trend continued to 22.6% (W5). This is still 5% higher than prepandemic but shows clear improvement in overall mental health over the course of 2022.

Similarly, after an increase in anxiety of children and adolescents aged 11–17 years during 2020 compared to prepandemic data (prepandemic: 14.9%, W1: 24.1%, W2: 30.1%), anxiety decreased in autumn 2021 (W3: 26.8%), slightly increased in Feburary 2022 (W4) to 27.8% and then decreased further to 24.8%, which is about the same level as at the start of the pandemic (W1, see Figure 3).

While depressive symptoms in adolescents aged 11–17 years initially only slightly, non-significantly increased between prepandemic data and W1 (CES-DC: 15.0 to 18.0%; PHQ-2: 10.0 to 11.3%), the increase reached significance in W2 (CES-DC: 24.3%, PHQ-2: 15.0%), followed by a significant decrease in Wave 3 (CES-DC: 19.7%, PHQ-2: 11.1%), a slight increase in W4 (CES-DC: 21.3%, PHQ-2: 12.8%), and finally a significant decrease in W5 (CES-DC: 14.2%, PHQ-2: 8.6%). However, most differences were small.

Girls had a greater risk of reporting both anxiety (RR ranged between 1.0 and 2.1) and depressive symptoms (RR ranged between 1.4 and 1.9).

### 3.4. Changes in psychosomatic complaints

**Figure 4:**
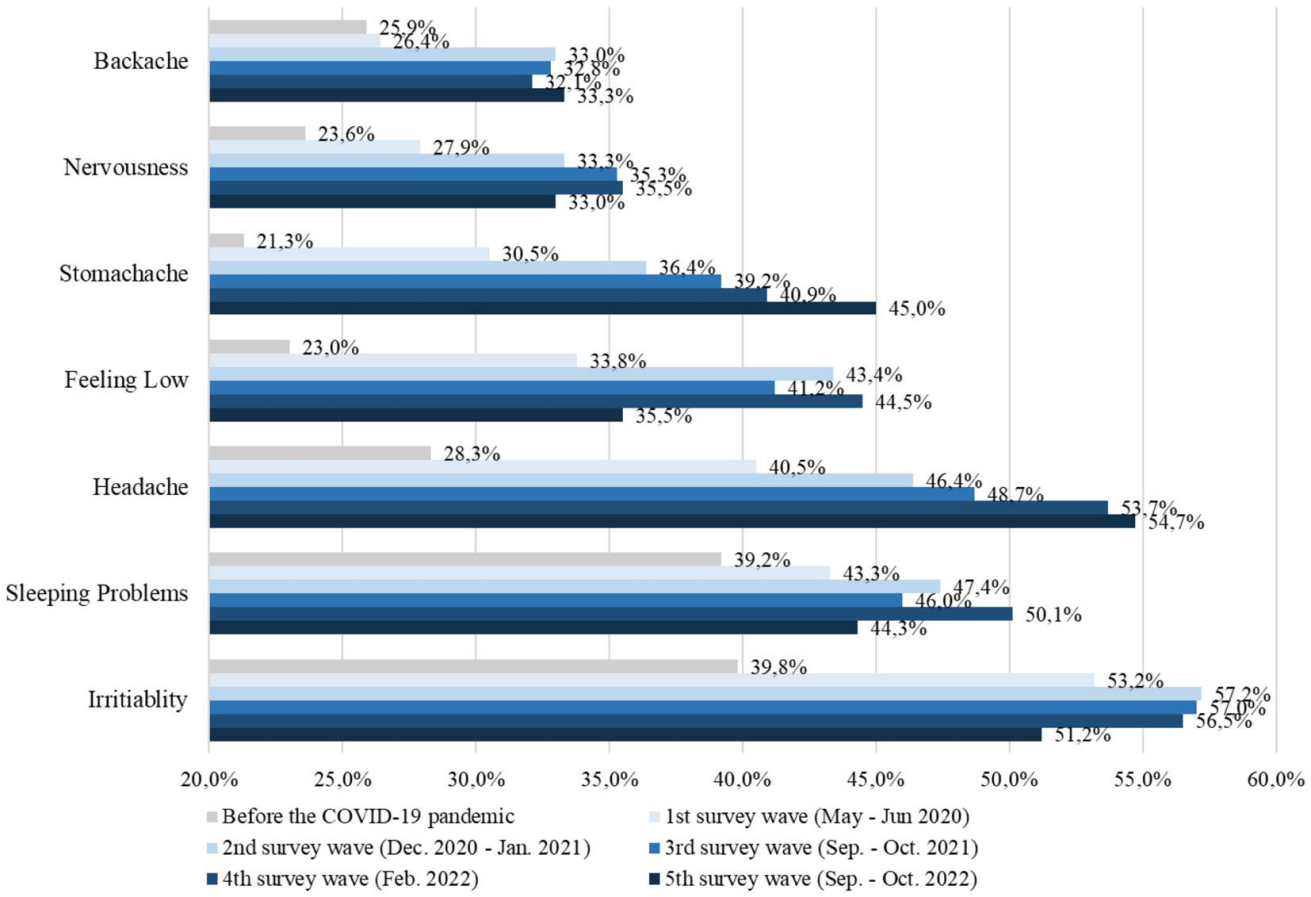
Psychosomatic complaints of children and adolescents aged 7–17 years old from 2020 to 2022.

Self-reported psychosomatic complaints in adolescents aged 11–17 years peaked between W1 and W3, depending on the specific complaint. Irritability, sleeping problems, feeling low and nervousness decreased in autumn 2022, but are still considerably higher than prepandemic. Headaches and stomachaches gradually increased from 2020 to 2022, i.e., about half of all children and adolescents suffered from them at least once during the past week. Back pain has remained on a higher than prepandemic level since W2 (W5: 33.3% vs. prepandemic: 25.9%). Further details of psychosomatic complaints can be found in Figure 4. Girls were at a higher risk of psychosomatic complaints in most complaints and waves, in particular with regard to having headaches (RR between 1.2 and 1.9) and feeling low (RR between 0.9 and 1.9).

Children and adolescents with a reported previous COVID-19 infection self-reported slightly higher prevalences of psychosomatic complaints. However, the differences were only statistically significant for some complaints (i.e., irritability as well as being impaired by these symptoms) (*V* = 0.10–0.14).

### 3.5. Risks and resources of children and adolescents’ HRQoL and mental health

The logistic regression analyses showed that at all five waves, children and adolescents aged 11–17 and belonging to the risk group had a higher risk (Odds Ratio, OR) of experiencing low HRQoL (ORs ranged from 2.4 to 3.3), mental health problems (ORs ranged from 2.7 to 5.1), anxiety symptoms (ORs ranged from 1.2 to 2.2), and depressive symptoms compared with their peers (ORs ranged from 2.4 to 3.7). The prevalence of having low HRQoL, and overall mental health problems was higher for the risk group across all 5 waves (see Table 2). Children and adolescents belonging to the resource cluster, on the other hand, had a significantly reduced risk (OR) of low HRQoL (OR ranged from 0.1 to 0.2), mental health problems (OR ranged from 0.1 to 0.2), anxiety symptoms (OR ranged from 0.2 to 0.3) and depressive symptoms (OR ranged from 0.1 to 0.2).

**Table 2:** Logistic regression of risks and resources on low HRQoL, mental health problems and symptoms of anxiety and depression (11–17 year olds).

		Low HRQol		Mental health problems		Anxiety symptoms		Depressive symptoms	
		OR	Sig	OR	Sig	OR	Sig	OR	Sig
Risk group	Wave 1	2.35	***	2.67	***	1.98	***	2.84	***
	Wave 2	3.32	***	2.96	***	2.21	***	3.05	***
	Wave 3	2.53	***	2.67	***	1.20		2.35	***
	Wave 4	3.02	***	4.71	***	2.81	***	3.65	***
	Wave 5	3.02	***	5.10	***	2.18	***	2.58	***
Resource cluster	Wave 1	0.15	***	0.21	***	0.28	***	0.18	***
	Wave 2	0.11	***	0.18	***	0.22	***	0.15	***
	Wave 3	0.10	***	0.15	***	0.27	***	0.13	***
	Wave 4	0.18	***	0.16	***	0.24	***	0.16	***
	Wave 5	0.07	***	0.10	***	0.24	***	0.09	***
Female	Wave 1	1.05		0.81		1.03		1.49	
	Wave 2	1.09		0.77		0.72		1.15	
	Wave 3	1.00		0.62		1.59	*	1.46	
	Wave 4	0.96		0.72		1.94	**	1.45	
	Wave 5	0.73		0.51	*	0.88		1.22	
Age 14–17	Wave 1	0.74		0.69		0.49	**	0.90	
	Wave 2	1.14		0.63		0.51	**	1.06	
	Wave 3	0.83		0.43	***	0.71		1.07	
	Wave 4	0.88		0.58	*	0.86		1.53	
	Wave 5	1.06		0.53	*	0.89		2.06	
Female * Age 14–17	Wave 1	1.48		0.96		2.50	**	1.06	
	Wave 2	1.13		1.37		4.19	**	1.48	
	Wave 3	1.60		1.63		1.11		0.98	
	Wave 4	1.01		0.94		0.74		0.77	
	Wave 5	1.37		1.15		1.42		0.76	
Nagelkerke’s R^2^	Wave 1	0.25		0.14		0.14		0.18	
	Wave 2	0.32		0.17		0.20		0.22	
	Wave 3	0.30		0.19		0.12		0.20	
	Wave 4	0.23		0.22		0.17		0.21	
	Wave 5	0.33		0.26		0.14		0.22	

Significance levels: *p < 0.05; **p < 0.01, ***p < 0.001.

Regarding age-specific differences, adolescents aged 14–17 had a lower risk of mental health problems at W3 through W5 compared with 11- to 13-year-olds (OR ranged from 0.4 to 0.6). Further, 14- to 17-year-olds had a lower risk of anxiety at W1 and W2 compared with 11- to 13-year-olds (ORs were 0.5, see Table 2). The logistic regression models fit the data well with the Hosmer and Lemeshow Tests both being non-significant and Nagelkerke’s Pseudo R^2^ ranging between 0.12 and 0.33.

Also, children and adolescents from single parent families had a significantly increased risk (OR) of impaired HRQoL in W1 (OR: 1.55) and W2 (OR: 1.39), for mental health problems (SDQ) in W3 (OR: 1.60), W4 (OR: 1.43) and W5 (OR: 1.41), depressive symptoms (CES-DC) in W2 (OR: 1.50) and W5 (OR: 1.48) and for anxiety symptoms (SCARED) in W1-W5 (OR: 1.64, 1.38, 1.64, 1.40, 1.43).

### 3.6. New worries of children and adolescents in 2022

**Figure 5:**
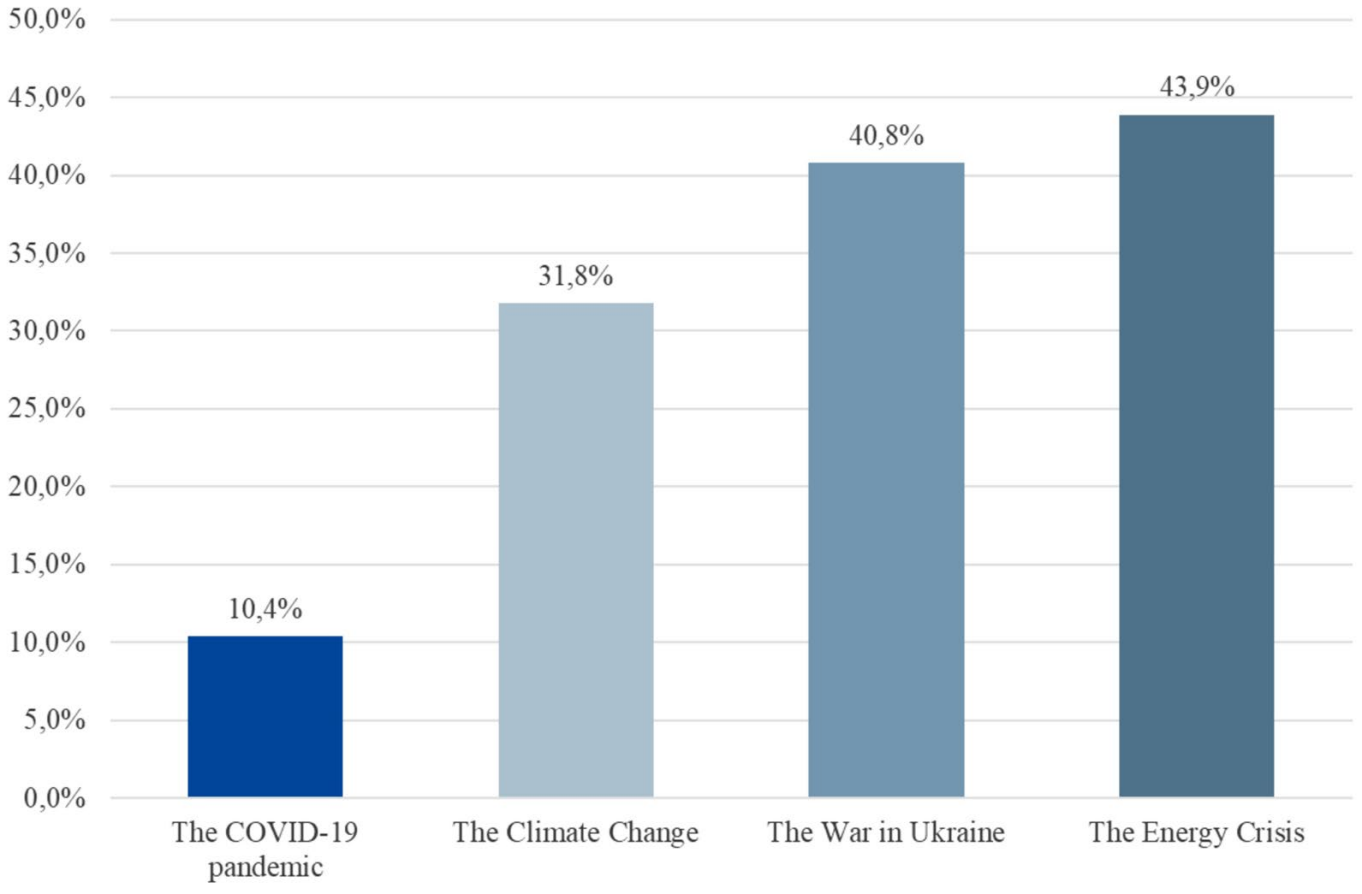
Worries of children and adolescents aged 11–17 years in autumn 2022.

Administering novel questions about current worries for the first time in autumn 2022, the COPSY study found that almost half of all children and adolescents aged 11–17 years old were *quite* concerned or *very* worried about the financial and energy crisis during the winter, and nearly as many of them indicated worries about the war in Ukraine. A third of the surveyed youth were also worried about the climate crisis, while only about 10% indicated to still worry about the pandemic (Figure 5).

Items assessing future anxiety in the context of current crises (like the COVID-19 pandemic, climate crisis, the war etc.) revealed that 82.6% of the youth often report the dominating fear that the current crises will remain for a long time. 78.4% reported fear that their life will get worse due to crises and 74.5% were worried that families will be able to afford less in the future. About half of the children (50.6%) were deeply worried about the uncertainty related to crises, e.g., they were afraid that they will not achieve their school qualification or professional goals in the future.

## 4. Discussion

The current data (COPSY W5) from autumn 2022 of the longitudinal COPSY study shows a specific pattern (in line with our first hypothesis): After two years of pandemic-related deterioration in child and adolescent mental health, an improvement has finally taken place in 2022. First slight improvements were already visible in autumn 2021, but leveled out in winter 2021/22. The changes between W4 and W5 of the COPSY study provide evidence that this trend of improvement occurred throughout the past year. However, except for depressive symptoms, which show a recovery effect to prepandemic levels, we surprisingly found that in year three of the pandemic most mental health outcomes are still worse than before the pandemic, and some self-reported psychosomatic complaints like headaches and stomachaches are still steadily on the rise.

The finding for the sharp initial increase of mental health problems at the beginning of the pandemic, which then stabilized at a high level from 2020 until spring 2021, is in line with the results of current literature reviews synthesizing the respective evidence during the first 1.5 years of the pandemic (2, 5, 6). Only one recent meta-analysis could not report clear mental health deteriorations during the pandemic, potentially due to comparing studies of young children with university students and comparing studies with small/non-representative samples (8). Most of the to-date published studies either report on prepandemic vs. pandemic effects or were administered between 2020 and spring 2021. To our knowledge, only three other longitudinal studies (20, 61–63) and two health insurance reports (64, 65) have published data on the mental health of children and/or adolescents over the course of 2021 and/or 2022.

Witte et al. (65) published German statutory health insurance data covering the years 2018 until end of 2021. According to their data, diagnosed mental health problems in children and adolescents between the ages of 10 and 17 increased during the pandemic. For girls, there was a particular increase of mood disorders from 18% to 23%, of anxiety disorders from 7% to 24%, and eating disorders from 33% to 54% (65).

In Austria, a four-wave longitudinal study on children aged 3 to 13 years old and using parent reports found that children’s mental health (internalizing problems, posttraumatic stress symptoms) was worse in December 2021 than in March 2020 (66). In Italy, a three-wave longitudinal study on 5- to 6-year-old children using parent reports found that between 10/2019 and 03/2020 and 10/2021 the depressive scores of a risk group increased from t1 to t2 and stayed stable until t3, while the depressive scores of the non-risk group first decreased from t1 to t2 and then increased again (61).

In a German cohort study, Schnetzer & Hurrelmann (62, 63) describe, similarly to the COPSY study, slight improvements in mental health in 2022 in 14 to 29-year-olds. From March to October 2022 the percentage of those feeling “stressed out” decreased from 45% to 41% and the percentage of those feeling listless decreased from 35% to 31%. Similarly, small to minor decreases were reported for the proportion of adolescents feeling exhausted (32% to 29%) and depressed (27% to 26%).

Thus far, hardly any other repeated/interval study has reported mental health recovery effects as found by the COPSY and the study from Schnetzer & Hurrelmann (62, 63) for children and adolescents during the pandemic. Small, potentially interim, improvements have been reported by Hawes et al. (20) in relation to the peak of infection rates in New York in 2020 – and by Zuccolo et al. (67) in Brazil, however the improvements found in emotional problems in winter 2020/21 did not continue over the course of 2021.

The COPSY study is one of the first studies to show that the mental health of children and adolescents improved in 2022. These improvements in mental health could be caused by an ongoing adaptation process during the past pandemic years, e.g., increasing resilience; the resumption of normal social, physical, and entertainment activities due to fewer restrictions; the availability of vaccinations, which make most disease courses less severe; and the fact that most of the children coped with the COVID-19 infection itself well.

While mental health is improving for almost all outcomes in the COPSY study over time, it is important to note that 14% to 27% of the youth still suffer impairments in health-related quality of life, depression, anxiety or other mental problems. Therefore, we cannot yet speak of a full recovery to prepandemic levels.

In terms of our second hypothesis that some children are at a higher mental health risk than others, we replicated our previous results as hypothesized (10): for example that particularly girls show higher HRQoL impairments and more depressive and anxiety symptoms, which was replicated across most COPSY waves. And adolescents aged 14–17 years had a lower risk of mental health problems across most waves compared with 7–10-year-olds. This age and gender effect should be taken into account in prevention and treatment programs for the youth.

The fifth wave of the COPSY study also replicated that children with high family and social support and good personal resources (45%) had a 4 to 14 times higher chance of better mental health outcomes than other children. In contrast, 16% of children and adolescents belonging to a risk group with a higher parental pandemic burden or parental mental health problems or low parental education or restricted living space and migration background had an up to 5 times higher risk of mental health problems. This is in line with other studies (13, 19, 22, 35, 36).

Further, and different from the overall positive trend, the COPSY study surprisingly found an ongoing steady increase in self-reported stomachaches and headaches across the past three pandemic years, with almost half of the children and adolescents reporting them at least once per week. Other studies on somatic complaints in children and adolescents during the pandemic also report increases in somatic complaints. Bantel et al. (68) found an increase in headaches and stomachaches in children at the school entry level in a German town between prepandemic and pandemic assessments, and Hafstadt et al. (18) found an increase in overall somatic complaints in a three-wave representative Norwegian longitudinal study of 12- to 16-year-olds. However, the prevalence of headaches and stomachaches in the COPSY study is higher than the result of a large German school study (69), which found that only 23% of fifth- to tenth- graders had headaches and 14% had stomachaches at least once a week in 2020 and 2021. Whether the higher prevalence of stomachaches and headaches found in COPSY could be related to a higher rate of COVID-19 or other infections with such symptoms in the COPSY sample or could reflect higher strain in the COPSY sample being turned psychosomatic is an open question, which may be answered by future research. The hypothesis that those symptoms are psychosomatic, i.e., children who may not otherwise be able to express their worries or other mental problems may somatize them, may be a more likely hypothesis than those symptoms being directly related to a previous COVID-19 infection. More research should be initiated to further explore psychosomatic and somatic complaints of children during the pandemic.

And finally, as assumed in hypothesis three, while between 2020 and 2021 the COVID-19 pandemic was the topic children were most worried about, in 2022 new crises and future anxieties have emerged. Similarly to a longitudinal study conducted in 2022 in Germany (62) and as expected by us, the COPSY study shows that the pandemic is no longer the topic children and adolescents worry most about. The COPSY W5 found that children aged 11–17 years mostly worry about the energy or financial crisis, Schnetzer & Hurrelmann (62) also found that adolescents between the age of 14 to 29 years worry most about inflation. The second most worrisome topic for youth in both studies was the war in Ukraine, followed by climate change. When comparing the proportions of worried youth in these studies, adolescents aged 14–29 years appear more worried than the 11- to 17-year-olds in the COPSY study. This may be due to age-related differences in political interest and awareness, differences in media use, i.e., older children/adolescents may be more affected by crises, because they may be exposed to more news coverage on that or other factors like different methods of assessment in both studies.

Limitations of the COPSY study are that the sample was drawn by matching data from the German Microcensus (2018), so results may not be generalizable across countries; the survey was administered using an online panel and incentives were deployed, which may have influenced the participation. Lastly, the study does not allow the causal conclusion that the pandemic has caused all the results found; there could be other factors, like response bias, which may have caused them. Strengths of the COPSY study are the longitudinal design comprising almost 3 years, the large population-based sample, the availability of nationally representative, comparative prepandemic data, and the use of validated, internationally-established instruments.

## 5. Conclusion

Waves 4 and 5 of the COPSY study underline the trend of mental health improvements in 2022, but most mental health problems are still above the prepandemic baseline in year three of the pandemic. Some psychosomatic symptoms like self-reported headaches and stomachaches are on the rise. Only depressive symptoms recovered.

Mental health problems of minors, even if not (yet) clinically diagnosed, should not be overlooked because when untreated they can turn into mental disorders in adulthood causing prolonged suffering and an increase in health care costs.

For those mentally burdened children and adolescents who are still at risk, we recommend nationwide, low-threshold support. Such programs have also been recommended by the German Ethics Committee (70) and the Center for Disease Control (CDC) (71). In line with the German Ethics Committee, we call for support of those children and adolescents and their families to compensate for their many pandemic sacrifices. As an act of transgenerational solidarity, the German Ethics Committee is demanding now that our society focuses on the mental health care of minors (70).

Institutions that offer mental health diagnostics, counseling, treatment, and aids for the participation of youth and support of families need to be financed both reliably and long term. Improving mental health care seems particularly important in neighborhoods with limited living space, low education, financial problems, and a high percentage of migrants. Current severe deficits in mental health care need to be addressed and remedied. With the increasing demand for mental health care of youth, it is crucial to reduce waiting times for mental health care (72).

In view of current and future crises - like the Ukraine war and the climate crisis - and the demographic change with fewer younger people who have to bear more burdens compared to previous generations, it is crucial that existing burdens are reduced now to avoid long-term mental health impairments.

## Data availability statement

The raw data supporting the conclusions of this article will be made available by the authors, without undue reservation.

## Ethics statement

The studies involving human participants were reviewed and approved by Local Psychological Ethics Committee (LPEK-0151). Written informed consent to participate in this study was provided by the participants’ legal guardian/next of kin.

## Author contributions

UR-S, AK, ME, JD, FR, A-KN, MG, and AS contributed to conception and design of the study. LS organized the database. ME performed the statistical analysis. JD and UR-S drafted the first manuscript. ME, AK, JD, A-KN, MG, and LS wrote sections of the manuscript. All authors contributed substantially to the discussion of results, manuscript revision, read, and approved the submittedversion.

## Funding

We acknowledge financial support from the Open Access Publication Fund of UKE - Universitätsklinikum Hamburg-Eppendorf and DFG – German Research Foundation. The COPSY study was funded by the Jaekel Foundation and the Foundation “Wissenschaft in Hamburg.” Furthermore, the COPSY study is part of the NUM 2.0 project coverCHILD, funded by the Federal Ministry of Education and Research (BMBF). The funders had no role in study design, data collection and analysis, decision to publish, or the preparation of the manuscript.

## Conflict of interest

The authors declare that the research was conducted in the absence of any commercial or financial relationships that could be construed as a potential conflict of interest.

## Publisher’s note

All claims expressed in this article are solely those of the authors and do not necessarily represent those of their affiliated organizations, or those of the publisher, the editors and the reviewers. Any product that may be evaluated in this article, or claim that may be made by its manufacturer, is not guaranteed or endorsed by the publisher.
